# Persian Validation and Cultural Adaptation of the Body Dysmorphic Disorder Questionnaire-Aesthetic Surgery for Iranian Rhinoplasty Patients

**DOI:** 10.29252/wjps.10.2.55

**Published:** 2021-05

**Authors:** Hesam Jahandideh, Fatemeh Dehghani Firouzabadi, Mohammad Dehghani Firouzabadi, Ahmad Ashouri, Ali Haghighi, Maryam Roomiani

**Affiliations:** 1 ENT and Head & Neck Research Center, The Five Senses Health Institute, Iran University of Medical Sciences, Tehran, Iran.; 2 Department of Otolaryngology-Head and Neck Surgery, Firoozgar Hospital, Iran University of Medical Sciences, Tehran, Iran.; 3 Department of Clinical Psychology, School of Behavioral Sciences and Mental Health (Tehran Institute of Psychiatry), Iran University of Medical Sciences, Tehran, Iran.

**Keywords:** Body dysmorphic disorders, Rhinoplasty, BDDQ-AS, Screening tool, Iran

## Abstract

**BACKGROUND:**

Body dysmorphic disorder (BDD) is one of the obsessive-compulsive disorders (OCD) which is very common in populations. However, the diagnosis rate is lower than the reality. BDD may lead to loneliness, jobless, avoidance of daily life and public activities. The Body Dysmorphic Disorder Questionnaire-Aesthetic Surgery (BDDQ-AS) is the validated questionnaire used to screen the BBD in patients seeking cosmetic surgeries. This study aimed to translate and validate a Persian version of the BDDQ-AS.

**METHODS:**

This analytical-descriptive cross-sectional study was conducted at Firoozgar Hospital, Tehran, Iran to validate the BDDQ-AS in Iranian society in 2020. A standard forward and back-translation procedure was followed. Overall, 79 Persian-speaking patients of both sexes referred to rhinoplasty surgery department at Firoozgar hospital were recruited. The control group consisted of 70 patients who also completed the final questionnaire. The BDDQ-AS was translated into Farsi. The final version was tested for reliability and validity in both groups.

**RESULTS:**

The internal consistency and split-half test were 89.2% and 92% respectively in rhinoplasty group. The spearman`s correlation coefficient between the scores obtained in BDD-YBOCS and BDDQ-AS was 0.757 (*P*<0.001) which confirmed the criterion validity and the minimum value of CVI was 0.79 that all items were relevant, transparent and simple.

**CONCLUSION:**

The Persian version of the BDDQ-AS questionnaire consist of 6 short yes/no questions which is less time-consuming and reliable for interpreting and screening. The sensitivity and specificity of this version are 85.71% and 81% respectively, which are adequate for screening.

## INTRODUCTION

Facial and body appearance is an important feature for human beings that majority of people concerned about looking pleasing. Body dysmorphic disorder (BDD) is one of the obsessive-compulsive disorders (OCD) which is very common in populations. However, the diagnosis rate is lower than the reality^2^.

BDD is a well-defined psychiatric diagnosis which patients suffer from slight or perceived defect in physical appearance which is leading to significant impairment in important functioning area and affects their quality of life^3^. Any defects are exaggerated by the BDD patients and they feel embarrassment of their appearance. Some of the research suggested the higher prevalence rate of BDD in female and younger population which is seemingly related to the life experience and adaptation comes with age^4^. 

People who suffer from BDD spend many hours in front of the mirror to check their appearance commonly involve nose, eyes, skin, or hair. One of the most common cosmetic surgery among these patients is rhinoplasty. However, the consequence of the surgery is not satisfactory for them^5^. BDD may lead to loneliness, jobless, avoidance of daily life and public activities^6^. they usually suffer from important emotional distress such as anxiety and personality disorders^7^. The prevalence rate of BDD is 1%, 7% to 2.4% in general population. Comorbidities such as anxiety, depression, OCD, suicidal attempts are common among these patients^8^. Approximately 76% of patients with BBD consult for cosmetic procedures for their slight defects^7^. 

The diagnosis of BDD is challenging for surgeons and remains unknown to psychiatrics or psychologists. According to the poor satisfaction after surgery and aggression towards surgeons, it is important to identify patients with BDD in cosmetic procedures to select suitable treatment for them^7^. The common symptoms of BDD are negative emotional responses to the body parts^1^. 

The Body Dysmorphic Disorder Questionnaire-Aesthetic Surgery (BDDQ-AS) was the validated questionnaire used to screen the BBD in patients seeking for cosmetic surgeries^9^. The BDDQ-As is short questionnaire consist of seven items which sensitivity and specificity is 89.6% and 81.4% for rhinoplasty patients respectively^3^. This screening tool was developed in psychiatric setting but recently, was validated and used in facial plastic surgery setting^10^. 

The studies on BDD and the awareness of surgeons about this disorder have been growing recently. The results of another Iranian study showed the high rate of BDD (12.2%) in patients seeking for rhinoplasty^11^. However studies in other countries reported higher rates of BD. This discrepancy may be justified by the clinical interviews^12^. 

We aimed to translate and validate the Persian version of the BDDQ–Aesthetic Surgery in an aesthetic rhinoplasty population and tested it to estimate the prevalence and severity of BDD symptoms in the rhinoplasty candidates. Internal consistency, reliability and validity and sensitivity and specificity were also examined for this version. 

## MATERIAL AND METHODS

This analytical-descriptive cross-sectional study was conducted at Firoozgar Hospital, Tehran, Iran to validate the BDDQ-AS in Iranian society. Patients who were seeking for rhinoplasty were recruited at the plastic surgery of Firoozgar Hospital. Patients who had severe and very severe deficit in their nose were excluded from the study. Procedures were explained to the patients and written informed consent was obtained from all of them. The expert panel consist of psychiatric and psychologist applied for content validity. The translation, reliability and validity phases of the study were explained below.

***Translation***

Standard forward and back-translation procedure was followed. Two qualified translators conducted forward translations individually that were reconciled and merged. A third translator who is not aware of the content back-translated the corrected version for revision and identification of discrepancies. In 2020, seventy-nine Persian-speaking patients referred to rhinoplasty surgery department at Firoozgar hospital were recruited. The control group consisted of 70 patients who also completed the final questionnaire. Patients with severe defect were excluded from the study. 

The BDDQ-AS was derived from the BDDQ-Dermatology Version questionnaire with a little modification, to reduce the time and simplify the interpretation process for the aesthetic surgeon. This questionnaire is positive when patient was concern about the appearance and thinking about it (question 1 and 2) and also affect other sides of his/her life (more than 3 Yes response is considered positive BDDQ-AS). 

***Reliability***

The instrument was assessed through internal consistency and split test. Cronbach`s alpha was used to estimate the internal consistency. Statistical analysis of split test reliability was performed using Pearson’s correlation coefficient. 

***Validity***

The content validity was confirmed by expert panel and measured based on the Lashae and CVI formulas by Waltz and Basel in the Excel 2016 software. The reliability was determined by internal consistency and split-half method which the questions were divided into two parts, odd and even number, and the Spearman correlation coefficient was calculated their results. The content validity was examined by calculating the Content Validity Ratio (CVR) and Content Validity Index (CVI) according to opinions of 13 psychiatrics or psychologists who were asked to comment on the importance, relevance, transparency and simplicity of the translated items. The criterion validity was evaluated by concurrent validity which investigates the correlation between the results of the BDDQ-AS and the Brazilian-Portuguese versions of the Yale brown obsessive-compulsive scale modified for BDD (BDD-YBOCS) and construct validity was also examined by comparing known groups. 

***Statistical analysis***

Internal consistency of the questionnaire was defined by Cronbach`s alpha and for investigating the correlation between the quantitative variable, Pearson correlation coefficient was obtained. 

Data were analyzed by SPSS Statistics for Windows version 25.0 (Chicago, IL, USA) and MS Excel 2016. The minimum Cronbach`s alpha score was 0.7 to determine the internal reliability and the significance *P*-value level was less than 0.05. The minimum accepted Spearman score was 0.7 to confirm the correlation between the variables. 

***Ethical Approval***

All procedures performed in studies involving human participants were following the ethical standards of the institutional and/or national research committee and with the 1964 Helsinki declaration and its later amendments or comparable ethical standards. Informed consent was obtained from all individual participants included in the study.

## RESULTS

The range of age in both groups was 17 to 52 yr old. The demographic features of the patients in both groups is summarized in Table 1. There was no significance difference between rhinoplasty group and control group in age and gender. 

The reliability of the questionnaire was evaluated by Cronbach`s alpha and split-half test showed in Table 2. 

The calculated CVR and CVI values are shown in Table 3. The minimum accepted CVR value was 0.54 which all items except item number 6 got higher this point. The minimum value of CVI was 0.79 that all items were relevant, transparent and simple. The spearman`s correlation coefficient between the scores obtained in BDD-YBOCS and BDDQ-AS was 0.757 (*P*<0.001) which confirmed the criterion validity of this questionnaire and the mean scores of the BDD-YBOCS were significantly in the BDDQ-AS positive group than in the negative one.

 Based on the BDDQ-AS, 29 patients (36.7) from rhinoplasty group and 9 persons (12.8) from control group had body dysmorphic disorder which is significantly different between two groups. Therefore, the construct validity of this questionnaire was examined. Furthermore, there was no relation between age, sex, marital status and education with BDD. (Data not shown) 

**Table 1:** the demographic characteristic of rhinoplasty and control group

**Variable**	**Rhinoplasty group (n=79)** **N(%)**	**Control group (n=70)** **N(%)**	***P*** **-value**
**Age (yr) (mean, SD)**	27.96 (7.33)	25.7 (9.24)	0.703
**Male**	18 (22.7)	20 (28.5)	0.76
**Female**	61 (77.3)	50 (71.5)	
**Marital ( married)**	29 (37)	19 (27.1)	0.214

**Table 2:** the reliability of the translated BDDQ-AS questionnaire

**Test**	**Rhinoplasty group**	**Control group**
**Cronbach’s alpha**	0.892	0.881
**Split half**	0.92	0.881

**Table 3:** CVR and CVI for BDDQ-AS (n= 13 experts)

**Variable**	**Importance (CVR)**	**Relevance (CVI)**	**simplicity (CVI)**	**Transparency (CVI)**
**Item 1**	1	1	0.84	0.92
**Item 2**	0.84	0.92	0.92	0.84
**Item 3**	0.54	0.92	0.92	0.92
**Item 4**	0,84	1	0.92	0.92
**Item 5**	0.54	0.92	0.84	0.84
**Item 6**	**0.38**	0.84	0.84	0.92
**Item 7**	0.69	1	0.92	0.84

## DISCUSSION 

The BDD is a common issue among people who are seeking for the facial aesthetic surgery and therefore the cosmetic surgery has become increasingly popular. The prevalence of BDD in plastic surgery setting is significantly higher than general population (2.9% to 53.6%)^13^. The BDDQ-AS is a specific tool that evaluate the severity of BDD which is short and easy to use. 

In this study, general guidelines for cross-cultural adaptation were followed to develop a proper version of the BDDQ-AS in Persian (Appendix 1). Individuals who were recruited declared that the translated version was easy to understand. 

The Persian version of the BDDQ-AS was validated in sample of rhinoplasy surgery patients, showed excellent reliability (Cronbach`s alpha coefficient of 0.89 compared to 0.88 for the control group). 5.5% of 270 patients among a sample of orthodontic patients were screened positive with BDD-YBOCS questionnaire. In addition, the study showed BDD was more common in single females with younger age^14^. In that regard, other studies in cosmetic surgery settings indicate that more women seeking rhinoplasty^15^. However in this present study were not found any relation between age, sex and marital status with BDD. Another study was demonstrated BDD as equally distributed disorder across the world and sex^16^. 

The majority of patients who are seeking for cosmetic procedures and surgeries are female according to the previous study. In this present study, 77.3% of patients were female. These findings support the reports of another study of Iranian rhinoplasty patients^5^. Ramos et al showed high rate of BDD among cosmetic rhinoplasty patients. In their study, 80 rhinoplasty candidates were recruited which 50 of them showed distress related to the physical appearance. All participants were assessed by BDD-YBOCS and Body Dysmorphic Symptoms Scale (BDSS)^1^. 

The results of our study stated the high rate of BDD (36.7%) in rhinoplasty group. Rate of 12.2% have been found in another Iranian study by utilizing another screening instrument^11^. 

According to the CVR values item, number 6 was eliminated from the translated BDDQ-AS, but there was no significant difference in the results. The internal consistency and split half test were 89.2% and 92% respectively in rhinoplasty group which is consistent with The French validated BDDQ-AS questionnaire (Cronbach`s alpha was 0.90)^9^.

The tool should be compared against a similar instrument to assess the construct validity^17^. Thus, the BDDQ-AS was compared against the control group which declared significant differences in the results. The significant differences between two groups indicates that the BDDQ-AS was able to distinguish body dysmorphic disorders. 

This study was the first Persian version of the BDDQ-AS questionnaire which is useful for screening and monitoring patients. Patients with mild to moderate BDD can be satisfied from the outcome of rhinoplasty. Therefore, body dysmorphic disorder is not an exclusion criterion for cosmetic surgeries^18^. One of the limitations of this study is no finding of the severity of the BDD in patients. Another one is the small sample size.

## CONCLUSION

The Persian version of the BDDQ-AS questionnaire consist of 6 short yes/no questions which is really less time-consuming and reliable for interpreting and screening. The sensitivity and specificity of this version are 85.71% and 81% respectively, which are adequate for screening. This screening tool is validated and reliable to help surgeons to evaluate the outcomes of cosmetic rhinoplasty for patients.

## FUNDING

This research did not receive any specific grant from funding agencies in the public, commercial, or not-for-profit sectors. 

## References

[1] Ramos TD, de Brito MJA, Suzuki VY, Sabino Neto M, Ferreira LM (2019). High Prevalence of Body Dysmorphic Disorder and Moderate to Severe Appearance-Related Obsessive-Compulsive Symptoms Among Rhinoplasty Candidates. Aesthetic Plast Surg.

[2] de Brito MJ, Sabino Neto M, de Oliveira MF, Cordás TA, Duarte LS, Rosella MF (2015). Yale-Brown Obsessive Compulsive Scale modified for Body Dysmorphic Disorder (BDD-YBOCS): Brazilian Portuguese translation, cultural adaptation and validation. Rev Bras Psiquiatr.

[3] Lekakis G, Picavet VA, Gabriëls L, Grietens J, Hellings PW (2016). Body Dysmorphic Disorder in aesthetic rhinoplasty: Validating a new screening tool. Laryngoscope..

[4] Herbst I, Jemec GBE (2020). Body Dysmorphic Disorder in Dermatology: a Systematic Review. Psychiatr Q..

[5] Alavi M, Kalafi Y, Dehbozorgi GR, Javadpour A (2011). Body dysmorphic disorder and other psychiatric morbidity in aesthetic rhinoplasty candidates. J Plast Reconstr Aesthet Surg.

[6] de Brito MJA, Nahas FX, Cordás TA, Tavares H, Ferreira LM (2016). Body dysmorphic disorder in patients seeking abdominoplasty, rhinoplasty, and rhytidectomy. Plast Reconstr Surg.

[7] Joseph AW, Ishii L, Joseph SS, Smith JI, Su P, Bater K (2017). Prevalence of Body Dysmorphic Disorder and Surgeon Diagnostic Accuracy in Facial Plastic and Oculoplastic Surgery Clinics. JAMA Facial Plast Surg.

[8] Jawad MBM, Sjögren M (2017). [Body dysmorphic disorder]. Ugeskr Laeger..

[9] Milad D, Atallah M-R, Benamer Y-H, Saltychev M, Most SP, Moubayed SP ( 2019). French translation, cultural adaptation and validation of the BDDQ-AS for rhinoplasty patients. J Otolaryngol - Head Neck Surg [Internet].

[10] Dey JK, Ishii M, Phillis M, Byrne PJ, Boahene KDO, Ishii LE (2015). Body dysmorphic disorder in a facial plastic and reconstructive surgery clinic: measuring prevalence, assessing comorbidities, and validating a feasible screening instrument. JAMA Facial Plast Surg.

[11] Ghadakzadeh S, Ghazipour A, Khajeddin N, Karimian N, Borhani M (2011). Body Image Concern Inventory (BICI) for identifying patients with BDD seeking rhinoplasty: using a Persian (Farsi) version. Aesthetic Plast Surg.

[12] Vindigni V, Pavan C, Semenzin M, Granà S, Gambaro F, Marini M ( 2002). The importance of recognizing body dysmorphic disorder in cosmetic surgery patients: do our patients need a preoperative psychiatric evaluation?. Eur J Plast Surg [Internet].

[13] Metcalfe DB, Duggal CS, Gabriel A, Nahabedian MY, Carlson GW, Losken A (2014). Prevalence of Body Dysmorphic Disorder Among Patients Seeking Breast Reconstruction. Aesthetic Surg J [Internet].

[14] Yassaei S, Goldani Moghadam M, Aghili H, Tabatabaei SM (2014). Body dysmorphic disorder in Iranian orthodontic patients. Acta Med Iran.

[15] Bellino S, Zizza M, Paradiso E, Rivarossa A, Fulcheri M, Bogetto F (2006). Dysmorphic concern symptoms and personality disorders: a clinical investigation in patients seeking cosmetic surgery. Psychiatry Res.

[16] Ahluwalia R, Bhatia N, Kumar P, Kaur P (2017). Body dysmorphic disorder: Diagnosis, clinical aspects and treatment strategies. Indian J Dent Res.

[17] Gandek B, Ware JEJ (1998). Methods for validating and norming translations of health status questionnaires: the IQOLA Project approach. International Quality of Life Assessment. J Clin Epidemiol.

[18] Felix GAA, de Brito MJA, Nahas FX, Tavares H, Cordás TA, Dini GM (2014). Patients with mild to moderate body dysmorphic disorder may benefit from rhinoplasty. J Plast Reconstr Aesthetic Surg.

